# Inflammatory Pseudotumor of the liver mimicking peritoneal Carcinomatosis: a diagnostic challenge

**DOI:** 10.1093/omcr/omag185

**Published:** 2026-10-04

**Authors:** Ouiam Taibi, Zaynab Iraqi Houssaini, Rania Bouanane, Salma El Aouadi, Zineb Labbi, Omar El Aoufir, Laila Jroundi, Ola Messaoud

**Affiliations:** Department of Emergency Radiology, Avicenne Hospital, Mohammed V University, Avenue Hafiane Cherkaoui, Rabat 10000, Morocco; Department of Emergency Radiology, Avicenne Hospital, Mohammed V University, Avenue Hafiane Cherkaoui, Rabat 10000, Morocco; Department of Emergency Radiology, Avicenne Hospital, Mohammed V University, Avenue Hafiane Cherkaoui, Rabat 10000, Morocco; Department of Emergency Radiology, Avicenne Hospital, Mohammed V University, Avenue Hafiane Cherkaoui, Rabat 10000, Morocco; Department of Emergency Radiology, Avicenne Hospital, Mohammed V University, Avenue Hafiane Cherkaoui, Rabat 10000, Morocco; Department of Emergency Radiology, Avicenne Hospital, Mohammed V University, Avenue Hafiane Cherkaoui, Rabat 10000, Morocco; Department of Emergency Radiology, Avicenne Hospital, Mohammed V University, Avenue Hafiane Cherkaoui, Rabat 10000, Morocco; Department of Emergency Radiology, Avicenne Hospital, Mohammed V University, Avenue Hafiane Cherkaoui, Rabat 10000, Morocco

**Keywords:** inflammatory Pseudotumor, liver—Peritoneal Carcinomatosis- cirrhosis

## Abstract

Inflammatory pseudotumor of the liver is a rare benign entity with non-specific radiological features that can closely mimic malignant hepatic lesions, making its diagnosis challenging. We report the case of a patient in whom a focal hepatic lesion was detected on abdominal ultrasound, with imaging findings consistent with underlying cirrhosis. Subsequent contrast-enhanced computed tomography demonstrated a dysmorphic liver with infiltrative subcapsular hepatic masses, associated with micronodular infiltration of the peritoneal fat and cardiophrenic angle lymphadenopathy, raising strong suspicion for a malignant hepatic process with peritoneal carcinomatosis. The diagnosis of inflammatory pseudotumor was ultimately confirmed by a CT-guided percutaneous liver biopsy. This case emphasizes the importance of considering inflammatory pseudotumor in the differential diagnosis of hepatic lesions, as this entity may closely mimic malignant etiologies on imaging.

## Introduction

Inflammatory pseudotumors (IPTs) are rare benign lesions histologically characterized by a proliferation of inflammatory cells, including myofibroblasts and spindle cells [1]. Their etiology remains poorly understood, and the clinical presentation is often nonspecific. Diagnosis is challenging, as imaging findings are frequently non-specific and may closely mimic malignant hepatic tumors. We report a case of an indeterminate hepatic lesion in a cirrhotic-appearing liver, ultimately diagnosed as an inflammatory pseudotumor by image-guided biopsy.

## Case report

A 39-year-old woman with no known past medical history presented with right-sided low back pain associated with epigastric pain. She was afebrile, and physical examination revealed a tender hepatomegaly, with no other abnormal findings. No infectious trigger was identified, and the patient had no history of previous surgical or endoscopic interventions or known IgG4-related disease. Specific IgG4 testing was not performed.

Initial laboratory tests revealed mild liver enzyme abnormalities, with slight cytolysis and moderate elevations of alkaline phosphatase and gamma-glutamyl transferase. Total bilirubin, renal function parameters (urea and creatinine), and inflammatory markers were within normal limits. Serum tumor markers were not assessed.

As a first-line imaging modality, abdominal ultrasound was performed. It demonstrated a dysmorphic liver with irregular contours, right hepatic lobe atrophy, and compensatory hypertrophy of segment I and the left hepatic lobe, raising radiological suspicion of underlying cirrhosis. Multiple subcapsular hepatic lesions sharing similar imaging characteristics were identified, the largest being located in the right hepatic lobe. These lesions appeared hypoechoic, heterogeneous, and poorly defined. A small amount of abdominal ascites was also noted, with no other focal abdominal abnormalities (Fig. 1).

**Figure 1:**
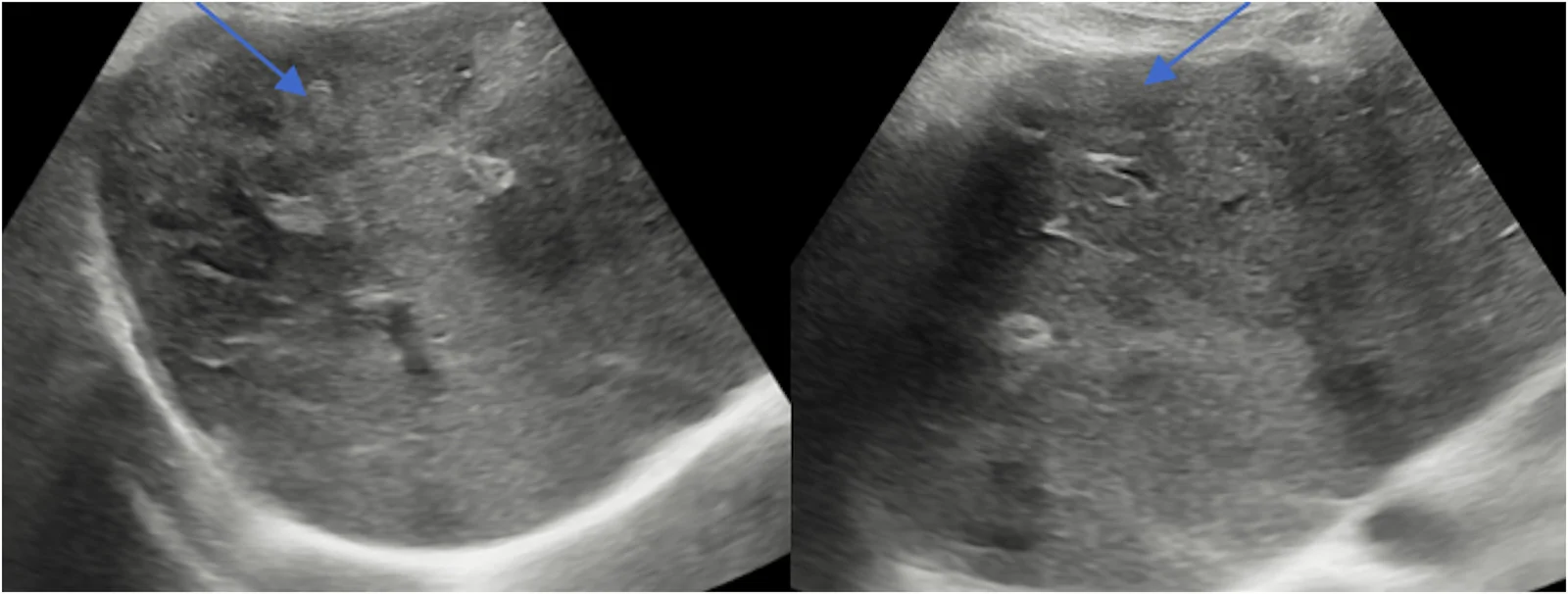
Abdominal ultrasound demonstrating a subcapsular hepatic lesion in the right hepatic lobe (arrow). The lesion appears hypoechoic, heterogeneous, and poorly defined.

Given these findings, a contrast-enhanced abdominal computed tomography (CT) scan was subsequently performed. In addition to the morphological features suggestive of underlying cirrhosis, CT revealed multiple infiltrative subcapsular lesions, predominantly involving the right hepatic lobe. The lesions appeared hypodense on non-contrast images and demonstrated heterogeneous enhancement after contrast administration, more pronounced during delayed phases, with central areas suggestive of necrosis (Fig. 2). The largest measured 149 × 53 × 139 mm and contacted the gallbladder and duodenum without clear invasion. It encased portal branches, which remained patent. Additional hepatic surface nodules, peri- and subhepatic peritoneal micronodules, mild ascites, and right cardiophrenic lymphadenopathy raised suspicion for peritoneal carcinomatosis (Fig. 3).

**Figure 2:**
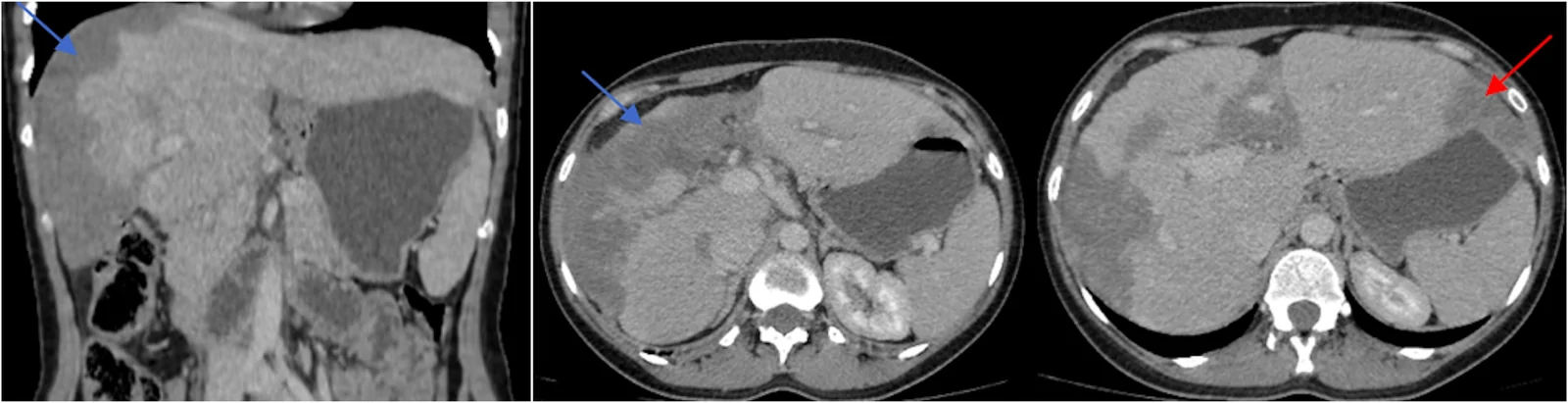
Contrast-enhanced abdominal CT scan showing a dysmorphic liver with irregular contours and compensatory hypertrophy of segment I. A large infiltrative subcapsular lesion in the right hepatic lobe (blue arrow) appears hypodense with heterogeneous post-contrast enhancement and central areas suggestive of necrosis. An additional hepatic lesion with similar imaging features is also observed (red arrow).

**Figure 3:**
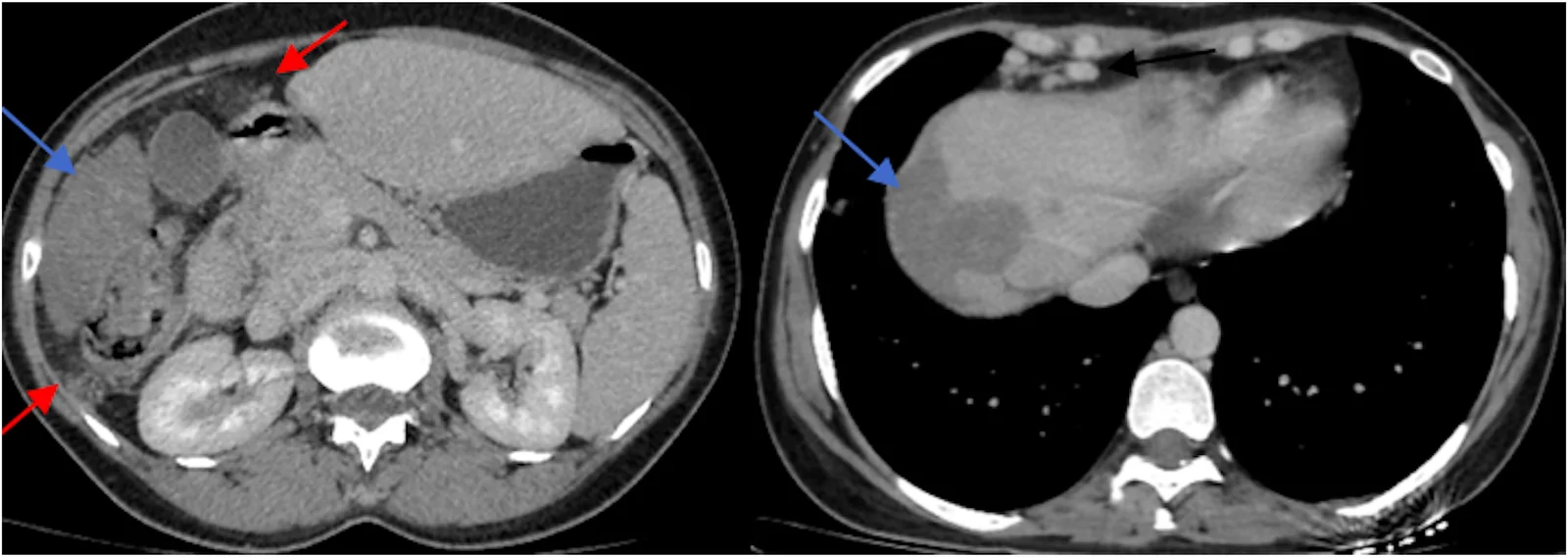
Contrast-enhanced CT demonstrating a hepatic lesion (blue arrow), micronodular peritoneal fat infiltration (red arrow), and right cardiophrenic angle lymphadenopathy (black arrow).

In view of the dysmorphic liver, infiltrative subcapsular hepatic lesions, peritoneal nodules, and right cardiophrenic lymphadenopathy, a malignant process with peritoneal carcinomatosis was strongly suspected, prompting liver biopsy.

Histopathology showed fibroblastic and myofibroblastic proliferation without cytological atypia within dense hyalinized stroma and a polymorphous inflammatory infiltrate. No malignancy was identified, and the findings were consistent with hepatic inflammatory pseudotumor (Fig. 4). As the biopsy was targeted to the hepatic lesion, the available specimen did not allow reliable confirmation or exclusion of underlying cirrhosis.

**Figure 4:**
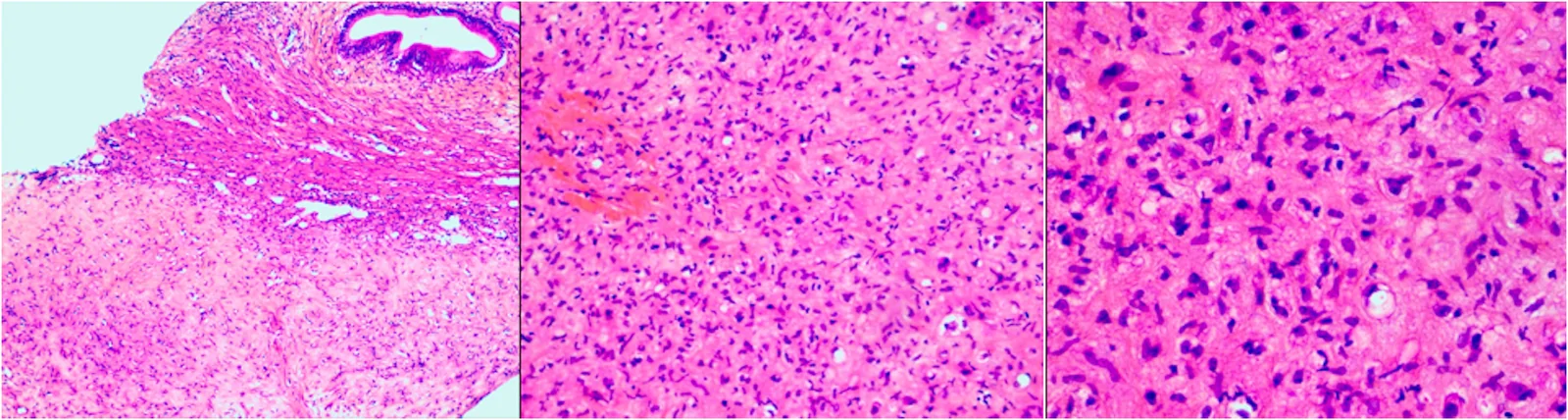
Histopathological examination showing hepatic parenchyma infiltrated by a fibroblastic and myofibroblastic proliferation composed of spindle-shaped cells with oval nuclei and no cytological atypia. The cells are embedded in a dense hyalinized fibrous stroma containing a polymorphous inflammatory infiltrate composed of lymphocytes, plasma cells, and polymorphonuclear leukocytes.

## Discussion

Inflammatory pseudotumors (IPTs) are benign lesions characterized by fibroblastic and myofibroblastic proliferation with a predominantly lymphoplasmacytic inflammatory infiltrate [2, 3]. They most commonly involve the lung, while hepatic involvement is rare [4, 5]. Hepatic IPTs remain diagnostically challenging because of their variable clinical and imaging features, often mimicking malignancy [6]. Few cases of hepatic IPT with omental or peritoneal involvement have been reported. Byun *et al*. described hepatic and right omental IPT associated with pelvic inflammatory disease [7], while Onieva-González *et al*. reported hepatic and omental lesions mimicking metastatic dissemination, with biopsies showing no malignancy [8].

The pathogenesis of hepatic IPT is not fully understood. Several mechanisms have been proposed, including infectious, autoimmune, and inflammatory processes, as well as associations with chronic hepatobiliary disease [3, 9]. Occasional cases have been reported in patients with chronic liver disease or cirrhosis, suggesting that long-standing hepatic inflammation may contribute to lesion development [3].

Clinical presentation is often nonspecific. The most common symptoms include abdominal pain and fever; however, approximately 15.6% of patients are asymptomatic [10]. Laboratory findings are equally variable and may show mild abnormalities in liver enzymes or inflammatory markers, while tumor markers are typically normal [11].

Imaging plays a crucial role in lesion detection; however, the radiological appearance of hepatic inflammatory pseudotumor is notoriously polymorphic and frequently mimics primary or metastatic hepatic tumors. On ultrasound, lesions are frequently hypoechoic and ill-defined. CT findings commonly include hypodense lesions with variable enhancement patterns, often demonstrating heterogeneous or delayed enhancement related to fibrotic components [12]. MRI features are also nonspecific, typically showing low signal intensity on T1-weighted images and heterogeneous high signal intensity on T2-weighted sequences, with delayed contrast enhancement in fibrotic areas [13]. In cirrhotic patients, diagnostic difficulty is further increased, as any atypical hepatic lesion remains suspicious for malignancy [14]. In our case, the infiltrative subcapsular growth pattern, delayed enhancement, central necrotic areas, peritoneal nodules, and cardiophrenic lymphadenopathy closely mimicked a malignant process with peritoneal carcinomatosis.

The differential diagnosis of hepatic inflammatory pseudotumor includes hepatocellular carcinoma, cholangiocarcinoma, metastatic disease, and lymphoma, as imaging findings often overlap with those of malignant hepatic tumors [15]. Peritoneal involvement and lymphadenopathy, as observed in this case, further complicate radiological interpretation.

Given these limitations, histopathological confirmation remains the cornerstone for definitive diagnosis [3].

Histological examination is essential for definitive diagnosis, demonstrating a fibroblastic and myofibroblastic proliferation embedded within a dense inflammatory infiltrate, without cytological atypia or features of malignancy [3]. Inflammatory myofibroblastic tumor is an important histopathological differential diagnosis, as it may overlap morphologically with inflammatory pseudotumor. Immunohistochemical assessment, particularly ALK testing, may help distinguish these entities [16]. In our patient, immunohistochemical analysis was not performed, representing a limitation of this case.

Image-guided biopsy is recommended for indeterminate lesions to establish diagnosis and avoid unnecessary surgery. Management of hepatic IPT is not standardized; conservative follow-up is appropriate after malignancy has been excluded, while some lesions may regress spontaneously or respond to anti-inflammatory or antibiotic therapy [6, 12, 16]. A major limitation of our case is the absence of clinical and radiological follow-up, as the patient was lost to follow-up after diagnosis, preventing assessment of treatment response and lesion evolution.
